# QueTAL: a suite of tools to classify and compare TAL effectors functionally and phylogenetically

**DOI:** 10.3389/fpls.2015.00545

**Published:** 2015-08-03

**Authors:** Alvaro L. Pérez-Quintero, Léo Lamy, Jonathan L. Gordon, Aline Escalon, Sébastien Cunnac, Boris Szurek, Lionel Gagnevin

**Affiliations:** ^1^UMR IPME, IRD-CIRAD-Université MontpellierMontpellier, France; ^2^UMR PVBMT, CIRAD-Université de la RéunionSaint-Pierre, France

**Keywords:** TAL effectors, phylogeny, *Ralstonia*, *Xanthomonas*, functional convergence, EBE

## Abstract

Transcription Activator-Like (TAL) effectors from *Xanthomonas* plant pathogenic bacteria can bind to the promoter region of plant genes and induce their expression. DNA-binding specificity is governed by a central domain made of nearly identical repeats, each determining the recognition of one base pair via two amino acid residues (a.k.a. Repeat Variable Di-residue, or RVD). Knowing how TAL effectors differ from each other within and between strains would be useful to infer functional and evolutionary relationships, but their repetitive nature precludes reliable use of traditional alignment methods. The suite QueTAL was therefore developed to offer tailored tools for comparison of *TAL* effector genes. The program DisTAL considers each repeat as a unit, transforms a TAL effector sequence into a sequence of coded repeats and makes pair-wise alignments between these coded sequences to construct trees. The program FuncTAL is aimed at finding TAL effectors with similar DNA-binding capabilities. It calculates correlations between position weight matrices of potential target DNA sequence predicted from the RVD sequence, and builds trees based on these correlations. The programs accurately represented phylogenetic and functional relationships between TAL effectors using either simulated or literature-curated data. When using the programs on a large set of TAL effector sequences, the DisTAL tree largely reflected the expected species phylogeny. In contrast, FuncTAL showed that TAL effectors with similar binding capabilities can be found between phylogenetically distant taxa. This suite will help users to rapidly analyse any *TAL* effector genes of interest and compare them to other available *TAL* genes and should improve our understanding of TAL effectors evolution. It is available at http://bioinfo-web.mpl.ird.fr/cgi-bin2/quetal/quetal.cgi.

## Introduction

Transcription activator-like (TAL) effectors are *Xanthomonas* proteins that are translocated into the plant cell through the type III secretion system and directed to the nucleus where they commandeer the cell metabolism by specifically activating plant genes (Bogdanove et al., 2010). In several pathovars they were demonstrated to be major aggressiveness determinants responsible for symptoms. In some situations they also act as avirulence factors, i.e., triggering the hypersensitive response notably when activating “executor” resistance genes (Boch and Bonas, 2010). Their mode of action has been detailed and their most outstanding feature is their central repeat domain, which is responsible for their highly specific attachment to DNA in regions known as EBE (effector binding elements). This domain contains 1.5–33.5 repeats of 33–35 amino acids. In each repeat the 12th and 13th amino acids are variable (therefore called “Repeat Variable Di-residue” or RVD) and dictate the specific interaction with a single nucleotide of the target DNA. Hence the successive RVDs in the protein are involved in specific attachment to a sequence of contiguous nucleotides located in the promoter of the gene to be activated. The correspondence between RVD and nucleotide, the “TAL code,” has been deciphered and demonstrated experimentally and may be used to predict targets of TAL effectors in plants (Boch et al., 2009; Moscou and Bogdanove, 2009). Researchers are now confronted by a wide array of potential TAL effector targets that can be experimentally explored to understand the mechanisms of *Xanthomonas* pathogenicity (through susceptibility targets), as well as some mechanisms underlying plant resistance to *Xanthomonas* (through executor resistance genes). Eventually this may help to develop new tools to breed resistant plants, either by escaping susceptibility or by introgressing executor resistance genes (Bogdanove et al., 2010; Boch et al., 2014).

As more TALomes, i.e., repertoires of *TAL* effector genes, are discovered and sequenced, one challenging issue has been to classify and compare them in order to (1) understand phylogenetic relatedness between TAL effector genes and decipher their modes of evolution; (2) assess functional similarities between TAL effectors and predict cases of functional convergence.

Alignment and distance calculation between *TAL* effector genes at the DNA or protein level are not straightforward due to the high sequence similarity between repeats, which are often identical over the majority of their sequence, with few variable residues not providing enough weight to correctly align orthologous repeats. To avoid this problem several works use alignments of the N-terminal and/or C-terminal regions of TAL effectors (Bogdanove et al., 2011; Yu et al., 2011; Pereira et al., 2014). However, sequences for these regions are not always available because sequencing efforts usually concentrate on the central repeat region which is more useful for functional studies. Furthermore, the distal regions are highly similar and may not allow discriminating between genes. In addition, the evolution and diversification of *TAL* effector genes may rely heavily on duplication and recombination, which is facilitated by their frequent localization on mobile insertion cassettes (MICs) (Ferreira et al., 2015) and their repeated structure (Lau et al., 2014). This produces multiple paralogous copies of similar genes sequences differing through insertion, deletion or reshuffling of their repeat units.

Currently, there is no systematic way to predict similar DNA binding capabilities among TAL effectors, other than through comparison of outputs from TAL effector binding site prediction software (Noel et al., 2013; Booher and Bogdanove, 2014). This turns to be impractical when dealing with large sets of sequences particularly when different species and pathovars are involved or through visual comparisons of RVD sequences, which in addition to being unworkable leaves out the variable binding inherent in the RVD-DNA code.

In this paper we describe two methods, DisTAL and FuncTAL, to align and classify *TAL* effector gene sequences according to their central repeat region. With DisTAL, we propose a tool that infers phylogenetic relationships between genes by considering each repeat as a unit and calculating distances between arrays of repeats, using an algorithm initially designed to compare microsatellite sequences (ARLEM version 1.0, Abouelhoda et al., 2010). FuncTAL aims to find functionally-related TAL effectors by calculating similarities in binding probabilities according to the RVD-DNA code. Together, these programs will help researchers infer evolutionary and functional relationships within and between groups of TAL effectors.

## Materials and methods

### Datasets

The sequences of 229 TAL effectors were obtained from the NCBI protein and nucleotide databases (http://www.ncbi.nlm.nih.gov/; accession numbers in Supplementary Table 1). This set was used for all analyses unless indicated and is referred to as the public dataset. 496 additional sequences (awaiting publication) were provided by collaborating laboratories, including those reported in Wilkins et al. (2015). These, together with public TAL effectors sequences are referred to as the full dataset. The species composition of the full dataset is found in Supplementary Table 2.

### Program specifications

FuncTAL and DisTAL are implemented in the Perl and R programming languages, they use the Perl modules Statistics::R, Bio::Perl (Stajich et al., 2002), and the R library APE (Paradis et al., 2004).

DisTAL additionally uses the module Algorithm::NeedlemanWunsch (http://search.cpan.org/~vbar/Algorithm-NeedlemanWunsch-0.03/lib/Algorithm/NeedlemanWunsch.pm) to align repeats and the C++ program ARLEM version 1.0 (Abouelhoda et al., 2010) to align sequences of coded repeats. Penalty parameters values for NeedlemanWunsch alignments are gap: 0, mismatch: −1, match: +1, alignment scores are normalized by dividing the score by the maximum length among the analyzed sequences and multiplying by 100 so they can be used by ARLEM. Parameters values for ARLEM are: align = TRUE, -insert = TRUE, ARLEM alignment scores are divided by 100 (so they can be used to build trees). Neighbor-joining trees are generated using the nj function of the package APE with default parameters (Paradis et al., 2004). The input file for DisTAL is a FASTA file containing amino acid sequences of TAL effectors. An additional file containing information on the TAL effectors can be used to color code the trees generated by the program. The following parameters can be modified: layout of the output tree (default = unrooted), include and compare input to TAL effectors from the public dataset (default = false), number of similar TAL effectors to output if the public database option is active (default = 5), exclude RVDs from analysis (default = false). Additional parameters can be modified in the standalone version: ARLEM indel penalization (default = 10), ARLEM duplication penalization (default = 10), and Create repeat distance matrix *de novo* (default = false). The outputs generated by DisTAL are:

- a pseudo-FASTA file (*Outputname*_CodedRepeats.fa) with the TAL effectors coded as a string of numbers,
- the set of unique repeats in the input file and their number codes (*Outputname*_RepeatsCode.txt)
- a matrix (*Outputname*.mat) showing the distances between the TAL effectors,
- a tree file (*Outputname*.tre) in Newick format to be used in any tree visualization program,
- a hits file (*Outputname*.hits) if the option to compare to a database was used; this file shows, for each TAL effector, the closest matches in our database.

DisTAL took an average of 0 m 22.3 s to process 200 TAL effector sequences in a computer with a Linux operating system with 15.6 Gb of RAM and an Intel® Core™ i7-4600U CPU @ 2.10 GHz processor. When the option to compare against public TAL effectors is activated, time goes up to 0 m 35.5 s.

FuncTAL uses modified subroutines (readMotifFile, compMotifs, scoreComparison, correlation) from the program compareMotifs.pl from the Homer (Hypergeometric Optimization of Motif EnRichment) suite (Heinz et al., 2010). The program can take as an input a text file with RVD sequences in the format “>RVD_id < tab>HD-NN-HD….” or a FASTA file containing nucleotide or amino acid sequences of TAL effectors. If a FASTA file is entered, the program will first recognize repeats in the TAL effector sequence as described for DisTAL. For each repeat the program next extracts the RVDs, i.e., the 12th and 13th amino acid (e.g., NN-HD). If the 13th amino acid is missing, as is the case for some repeats, the program inserts an asterisk “^*^.” Neighbor-joining trees are generated using the nj function of the package APE with default parameters (Paradis et al., 2004). The following parameters can be modified: layout of the output tree (default = unrooted), include and compare input to TAL effectors from the public dataset (default = false), and number of similar TAL effectors to output if the public database option is active (default = false). The outputs generated by FuncTAL are:

- a text file (*Outputname*.cons) showing the theoretical most likely binding site for each TAL effector,
- a matrix (*Outputname*.mat) showing the distances between the TAL effectors,
- a tree file (*Outputname*.tre) in Newick format to be used in any tree visualization program,
- a hits file (*Outputname*.hits) if the option to compare to a database was used, this file shows, for each TAL effector, the closest matches in our database according to their binding sites.

FuncTAL took an average of 1 m 22.3 s to process 200 TAL sequences in a computer with a Linux operating system with 15.6 Gb of RAM and an Intel® Core™ i7-4600U CPU @ 2.10 GHz processor. When the option to compare against public TAL effectors is activated, time goes up to 7 m 48.5 s.

The script used for simulated evolution of TAL effectors (Evolve.pl) is also made available at http://sourceforge.net/projects/quetaleffectors. This program uses the dist.topo function of the program APE (Paradis et al., 2004) to calculate topological distances between trees using the Penny and Hendy method (Penny and Hendy, 1985). The topological distance is defined as twice the number of internal branches defining different bipartitions of the tips (Penny and Hendy, 1985). The distances were normalized by the number of nodes in a tree. In this way a distance of 0 means identical trees, and the maximum distance of 2 means completely different trees.

The version of DisTAL that uses only sequences of RVDs (DistTAL-OnlyRVDs.pl) is also made available at http://sourceforge.net/projects/quetaleffectors. This version extracts the 12th and 13th amino acid from each repeats and then uses the same method for DisTAL, possible alignment scores between RVDs using the Needleman-Wunsch algorithm are 0, 50, and 100, and Indel penalization for ARLEM is 100 (both amino acids are deleted). This version was not extensively tested, thus it is not included in the web version.

ClustalW (Larkin et al., 2007) alignements were made using Clustal 2.1 with default parameters in two steps: clustalw -ALIGN and clustalw –TREE.

Muscle (Edgar, 2004) alignments were made using version 3.8.31 with default parameters. Alignments for t-coffee (Notredame et al., 2000) were made using version 10.00.r1613 (-gapopen = −50, -gapext = 0). And MAFFT (Katoh et al., 2005) alignments used the version 7.123b (E-INS-i –ep 0 –genafpair –maxiterate 1000). Parameters were chosen to allow long gaps in alignments. Trees were generated from these alignments using the dist-align function from the “seqinr” R package (http://seqinr.r-forge.r-project.org/) and the nj function from the APE package (Heinz et al., 2010).

### Programs availability

Packages containing the scripts of the FuncTAL and DisTAL programs, as well as additional scripts used in this work are available for download from Sourceforge at http://sourceforge.net/projects/quetaleffectors. A web interface and the source code for the suite are also available at http://bioinfo-web.mpl.ird.fr/cgi-bin2/quetal/quetal.cgi. The web version was created in Perl cgi-bin with w3c recommendations for CSS level 3 and Html 5.0 http://www.w3.org/standards/webdesign/htmlcss.

## Results

### QueTAL: DisTAL, a program for the phylogenetic classification of TAL effector repeat regions

The overall strategy to compare TAL effectors based on the sequence of their central repeat region consists in considering each repeat as a separate unit, and comparing the TAL effectors according to the nature and order of these units. This strategy is based on the assumption that repeats are the evolutionary units of *TAL* effector genes and can be deleted and duplicated as a whole. This is supported by the sequence and structural features of TAL effectors (Deng et al., 2012; Mak et al., 2012), recent models of TAL effectors evolution (Ferreira et al., 2015), as well as by works indicating that TAL effectors' functional specificity can be modified by changing the sequence of repeats (Herbers et al., 1992; Boch et al., 2009; Streubel et al., 2012), and that deletions or duplications occur in nature or and may be responsible for change in virulence and aggressiveness (Vera Cruz et al., 2000).

**Figure 1:**
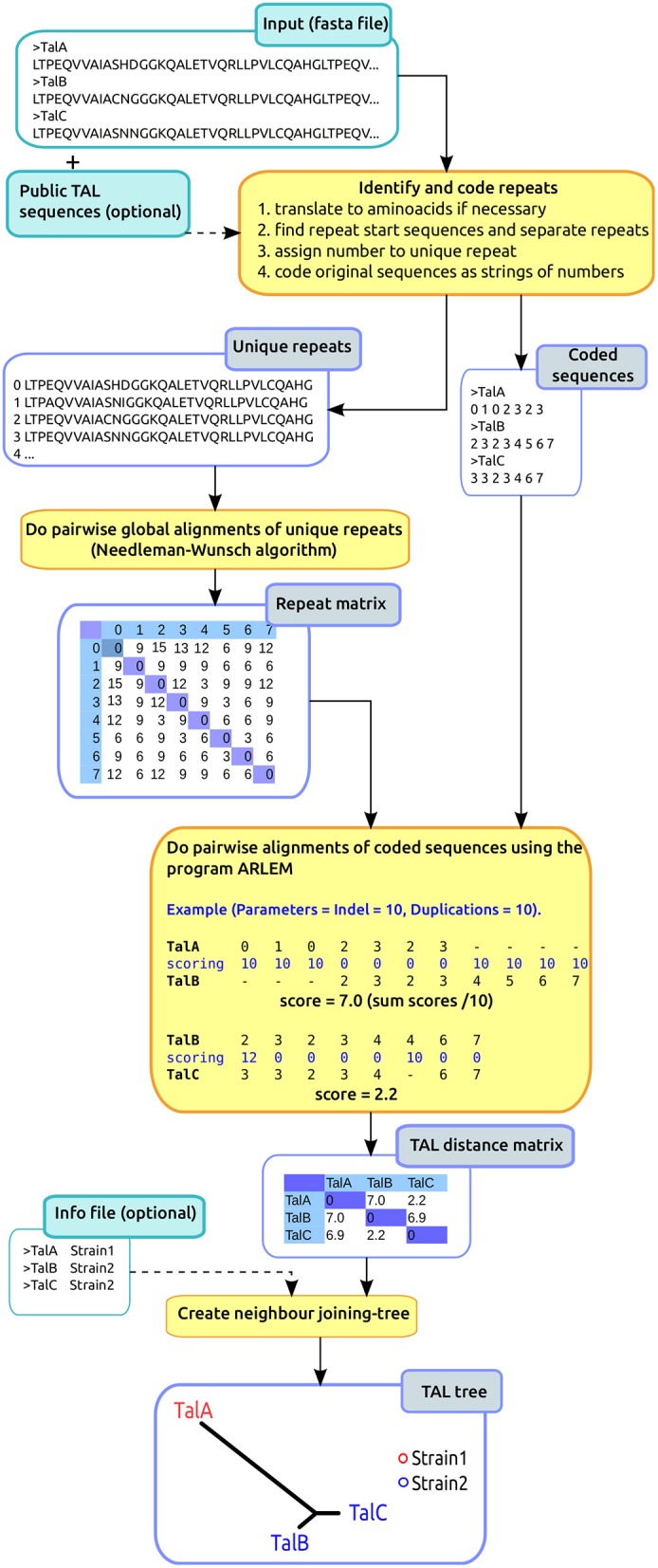
**DisTAL workflow**. The diagram shows a summarized version of the DisTAL workflow. An example is shown for 3 hypothetical TAL effectors from two strains containing 7–8 repeats each, and 10 unique repeats in total. Processes are shown in the orange squares, inputs (lighter blue) and outputs in blue squares.

To phylogenetically classify TAL effectors, we developed the program DisTAL, which classifies the input TAL effectors as a string of coded repeats and then uses the program ARLEM to calculate distances between these strings. The workflow for this program is depicted in Figure 1, and described next in detail.

#### Identification and coding of repeats

The program takes as input a set of TAL effectors to be analyzed, and if desired, the input TAL effectors can be compared to a dedicated database of 229 TAL effectors available in public DNA sequence databases (Supplementary Table 1). The input file should be a FASTA file containing either nucleotide or amino acid sequences of TAL effectors. If the input is nucleotide sequences these are translated to amino acids (in reading frame +1). It identifies and separates repeats in the input sequences by finding matches to motifs of 7 amino acids found at the start of repeats of known TAL effectors as traditionally defined (Boch and Bonas, 2010) (i.e., LTPDQVV). The program can also identify aberrant repeats (longer or shorter than average) and keep them for analyses. If they exist, the program also identifies and uses missing repeats (identified as strings of X's) which are sometimes included in TAL sequences due to sequencing gaps. It is however not recommended to include sequences with these gaps since these repeats will be assigned the maximum distance to any others.

Each unique repeat type is then assigned a numeric code and the original TAL effector sequences from the input file are transformed into sequences of coded repeats. Additionally the user can decide whether or not to exclude the RVDs from the analyses. If this option is chosen, the sequence analyzed for each repeat will be a concatenation of the 1st to 11th amino acid plus the amino acids from the 14th to the end of the repeat. This reduces the size and complexity of the repeat alphabet and, in theory, avoids biasing effects caused by different selection pressures acting on the RVDs.

#### Calculating distances between unique repeats

Next, a distance matrix is generated by calculating distances between every pair of unique repeats. For this, a global alignment with sliding ends (no gap penalty) is made for each pair of unique repeats using the Needleman-Wunsch algorithm (Needleman and Wunsch, 1970) as implemented in the Perl package Algorithm::NeedlemanWunsch (http://search.cpan.org/~vbar/Algorithm-NeedlemanWunsch-0.03/lib/Algorithm/NeedlemanWunsch.pm). The distances are normalized so that the repeat matrix information can be interpreted as the percentage of amino acids that change between repeats (based on the longest repeat among the two aligned). A distance matrix was generated for a set of 1110 unique TAL effector repeats found in our full dataset. It is included in the web version and the standalone version of the program to save computational time. If new repeats are found in the input file these are compared to the existing matrix and added to it.

Alternatively, the user can choose to generate this matrix using the Smith-Waterman (Smith and Waterman, 1981) algorithm for pairwise alignments as implemented in the Perl package Bio::Tools::pSW (http://search.cpan.org/dist/BioPerl/Bio/Tools/pSW.pm) using different amino acid substitution matrices (PAM30, PAM50, and Blosum62). This strategy is so far only available in the standalone version and it has not been extensively tested, however the results obtained with either matrix are often similar; the average topological distances for 50 trees obtained from 10 randomly selected TAL effector sequences when comparing the trees obtained with the Needleman-Wunsch algorithm to those obtained using Smith-Waterman + PAM30, PAM50, and Blosum62 were 0.36, 0.42, and 0.27, respectively.

#### Aligning and calculating distances between strings of coded repeats

To compare the sequences of coded repeats DisTAL uses the program ARLEM (also referred to as WAMI) (Abouelhoda et al., 2010) which was designed to compare minisatellite maps. A minisatellite map is a sequence of symbols that represents tandem arrays of short repetitive DNA segments such that the set of symbols is in one-to-one correspondence with the set of distinct repeats (Abouelhoda et al., 2010). We propose that, like minisatellites, TAL effector repeats when considered as evolutionary units can undergo three non-mutually exclusive processes: *Unit mutation* (change from one repeat to another), *duplication* (tandem copies of a repeat), and *insertion/deletion* (indels = loss or gain of new repeats) as described in Abouelhoda et al. (2010). In ARLEM each of these events is assigned a cost when aligning the sequences of units (Abouelhoda et al., 2010). In our case, the cost of unit mutation would be defined by the distance matrix generated in the previous steps, that is, the penalization for changing one repeat to another depends on the percentage of amino acids that are different between said repeats. The duplication and indel penalization is 10 for both events by default (a penalization equivalent to changing 10% of the amino acids from one repeat to another). These values were estimated by optimizing the length and score of sample alignments as shown below.

The alignment scores outputted by ARLEM for each pair of TAL effectors are the sums of the penalization values for mismatches, indels and duplications; the scores are then divided by 100. Consequently two TAL effectors with identical repeats will get an alignment score of zero. In contrast, two TAL effectors of the same length (i.e., 15 repeats), aligned with no gaps, and where each pair of aligned repeats differ from each other in 50% of the amino acids will have a score of 50 × 15/100 = 7.5.

#### Creating trees of TAL effectors

The scores outputted by ARLEM are organized into a matrix, which is then used to create a neighbor-joining tree using the R package APE (Heinz et al., 2010) in a user-defined format. The tree tip labels can be colored using an additional input file from the user that contains the TAL effector IDs and categories used to group them (e.g., species or strain).

### Optimizing the values of DisTAL parameters

**Figure 2:**
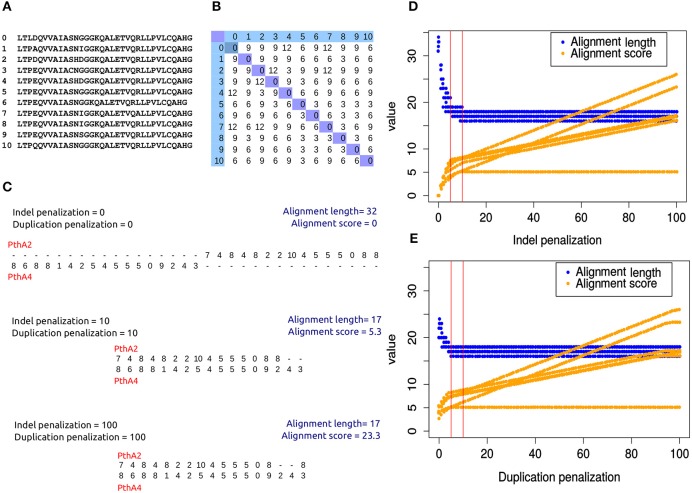
**Indel and duplication penalization for DisTAL**. The effect of different penalization values was assessed by generating alignments for 4 TAL effectors from strain IAPAR 306 of *Xanthomonas citri* pv *citri*. **(A–C)** Alignments between PthA2 and PthA4 are shown as an example. **(A)** Unique TAL repeats found in PthA2 and PthA4. **(B)** Scoring matrix between repeats as calculated by DisTAL using the Needleman-Wunsch algorithm. **(C)** Visualization of alignments of the coded sequences of PthA2 and PthA4. **(D)** Variation in alignment length and score for TAL effectors from *Xcc* IAPAR 306 using Distal with different indel penalization values. Duplication penalization was kept as 100, each point represents a pairwise alignment between two TAL effectors, red lines indicate range between 5 and 10. **(E)** Variation in alignment length and score for TAL effectors from *Xcc* IAPAR 306 using Distal with different duplication penalization values. Indel penalization was kept as 100. Alignment scores were multiplied by 10 for scaling.

To establish adequate penalizations for indels and duplications we optimized the alignments for the TAL effectors PthA1, PthA2, PthA3, and PthA4 from the *X. citri* pv. *citri* strain IAPAR 306 (Da Silva et al., 2002) since these TAL effectors are closely related (Pereira et al., 2014). When aligning the coded sequences for these TAL effectors, too many gaps are introduced if no penalizations for indels or duplications are used, thus resulting in very long alignments where the sequences may not even overlap. In contrast, using penalization values for indels and duplications that are too high results in alignments with fewer gaps but those gaps increase highly the alignment score (Figures 2A–C). As a consequence, the difference in score between gapped and ungapped alignments increases (Figures 2C,D), which could result in biased trees where TAL effectors with the same number of repeats tend to be grouped together.

We used DisTAL to run all pairwise alignments between these four *X. citri* pv. *citri* TAL effectors using different penalization values for indels and duplications, and looked for values that produced short alignments with little variation in the alignment scores for different TAL effector pairs. When keeping a high duplication penalization (100) and changing the indel penalization, the best alignments were found for penalization values between 5 and 10, with 10 producing shorter alignments (Figure 2D). Likewise when keeping a high indel penalization of 100 and changing the duplication penalization, the best alignments were found for penalization values between 5 and 10 (Figure 2E). The same results are found when changing both penalizations simultaneously (Supplementary Figure 1). Similar results were obtained using TAL effectors from the *X. oryzae* pv. *oryzae* (*Xoo*) strain PXO99^A^ and those from *X. oryzae* pv. *oryzicola* (*Xoc*) strain BLS256 (Supplementary Figure 2). The default value for both penalizations was then decided as 10.

### DisTAL accurately recreates the phylogeny of *In silico*-evolved TAL effectors

**Figure 3:**
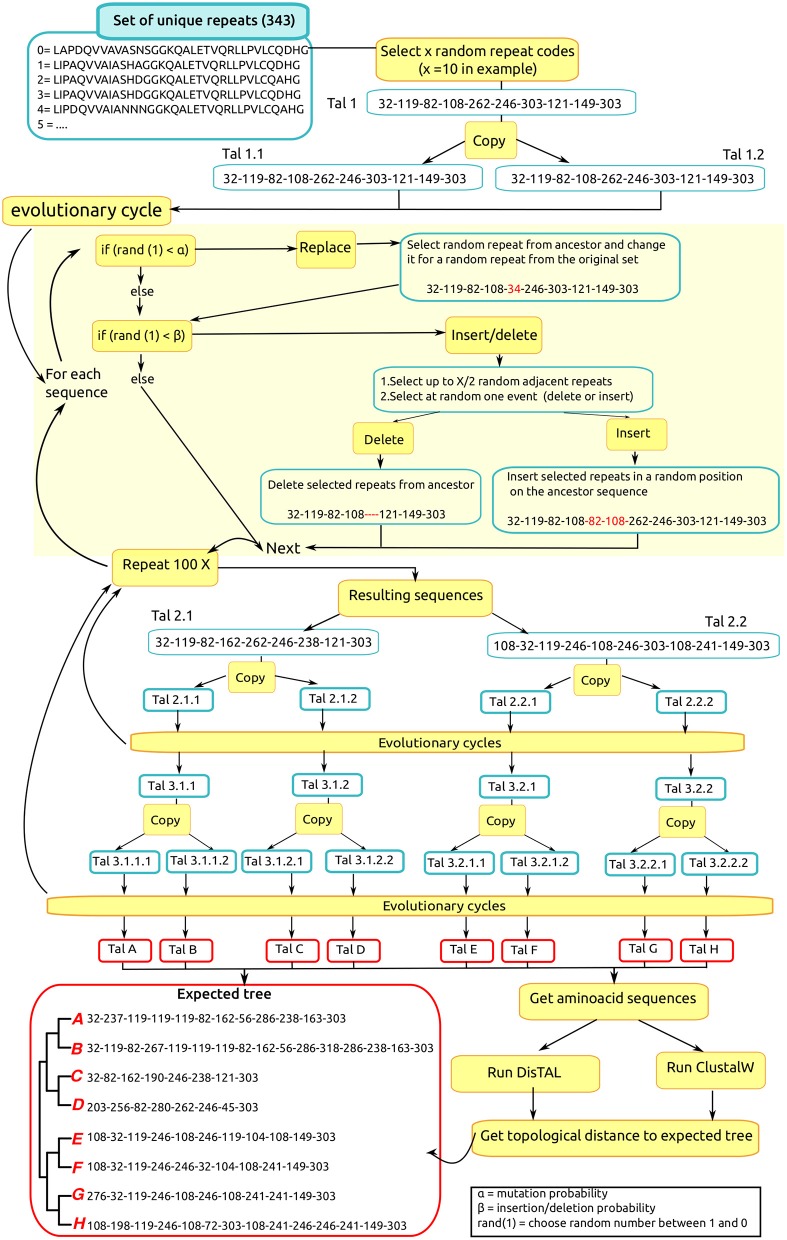
***In silico* evolution of hypothetical TAL effectors**. A hypothetical TAL effector is created and “evolved” as shown in the workflow for an example of a TAL effector with 10 repeats. α, replacement probability; β, indel probability; rand, randomly generated number between 0 and 1; X, TAL effector length in number of repeats. Intermediate TAL effectors in the process are named with numbers, the first number indicates generation (it increments after each evolutionary cycle) and subsequent numbers indicate descendance: when a TAL effector is copied, a number (0.1 or 0.2) is added to the name.

To test the ability of DisTAL to decipher the phylogeny of TAL effectors, we designed a script to simulate the evolution of TAL effectors under the assumption of repeats acting as evolutionary units (Figure 3). For this, an initial hypothetical TAL effector is created by randomly selecting 10 repeats out of a set of 344 unique repeats found in *Xoo* TAL effectors in the public dataset. Two copies (descendants) are then generated from this TAL effector and each descendant undergoes 100 evolutionary cycles where in each cycle two different events can occur:

**(i) Replacement:** a repeat is chosen at random and replaced by another one from the set of 344 repeats. This process is equivalent to mutating a series of amino acids in one repeat, the probability of this occurring in each cycle is designated α.

**(ii) Insertion/deletion:** a series of X (X = random value from 0 to 3) contiguous repeats are selected in the parent sequence and they have an equal probability of either being deleted, or being inserted into a random position in the TAL effector. The probability of this event occurring in each cycle is designated β. Note that this event also produces tandem duplications when the repeats are inserted next to their original position.

After 100 cycles the resulting two sequences are duplicated to produce a total of 4 descendants that each undergo the same process again. Finally, eight TAL effector sequences (named A–H) are produced from the initial TAL effector. We expect that a phylogenetic tree of these eight sequences should have this grouping as shown in Figure 3: [((A B)(C D))((E F)(G H))].

**Figure 4:**
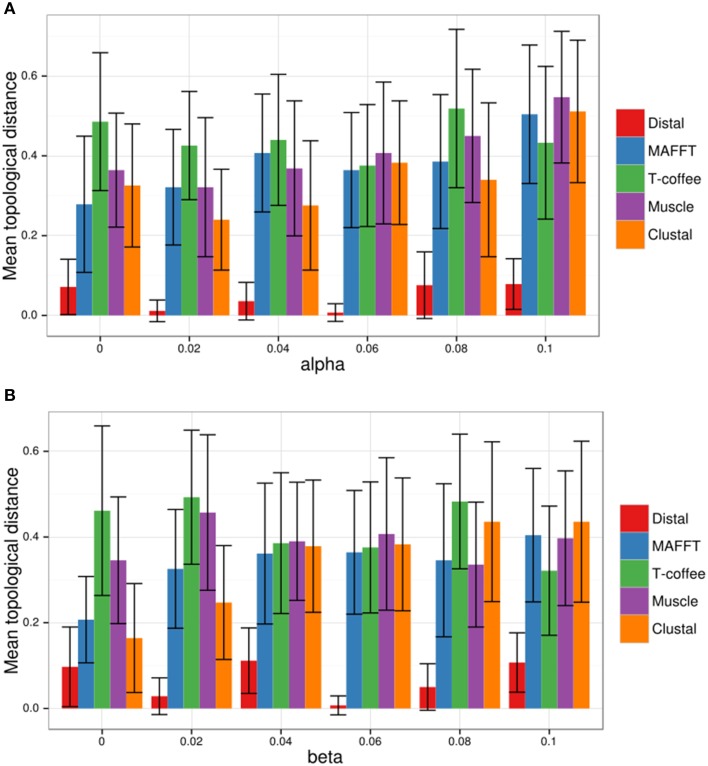
**DisTAL performance with *in silico*-evolved TAL effectors**. Sets of eight TAL effectors (named A–H) resulting from simulated evolution were fed into DisTAL, ClustalW, MAFFT, Muscle and T-coffee, the resulting trees were compared to the expected tree [((A B)(C D))((E F)(G H))], the scatter plot shows the topological distance. **(A)** Different values of alpha (probability of repeat replacement) were used to generate the sets of TAL effectors while keeping beta (probability of repeat indel) at a value of 0.06. **(B)** Different values of beta were used to generate the sets of TAL effectors while keeping alpha at a value of 0.06. Each bar represents the average topological distance for 100 sets of TAL effectors, error bars indicate standard deviation.

Next the resulting eight TAL effectors were fed into DisTAL (under default parameters with duplication and indel penalties equal to 10) and the resulting tree was compared to the expected tree. The topological distance between the trees was calculated using the Penny and Hendy method (Penny and Hendy, 1985), as implemented in the R package APE (Paradis et al., 2004). As shown in Figure 4, this process was repeated 100 times for different combinations of α and β values, from 0 to 0.1 with 0.005 increments (40,000 trees total), to account for different evolution scenarios. DisTAL consistently produced trees that differed little from the expected tree (mean topological distance = 0.09, median = 0). The program worked better when α and β were both higher than 0.02 (at zero all the TAL effectors have the same distance and all the nodes are at the same distance), and slightly better when β was higher than α. The trees obtained with DisTAL were also compared to trees obtained by doing multiple alignments of the repeat regions of the simulated TAL effectors using the programs for multiple alignment ClustalW (Larkin et al., 2007), MAFFT (Katoh et al., 2005), Muscle (Edgar, 2004), and T-coffee (Notredame et al., 2000) and then generating neighbor-joining trees. DisTAL consistently produced trees with closer resemblance to the expected tree than those obtained after alignment with other multiple alignment programs (Figure 4, Supplementary Figure 3).

### QueTAL: FuncTAL, a program for comparison of TAL effectors based on DNA binding specificities

**Figure 5:**
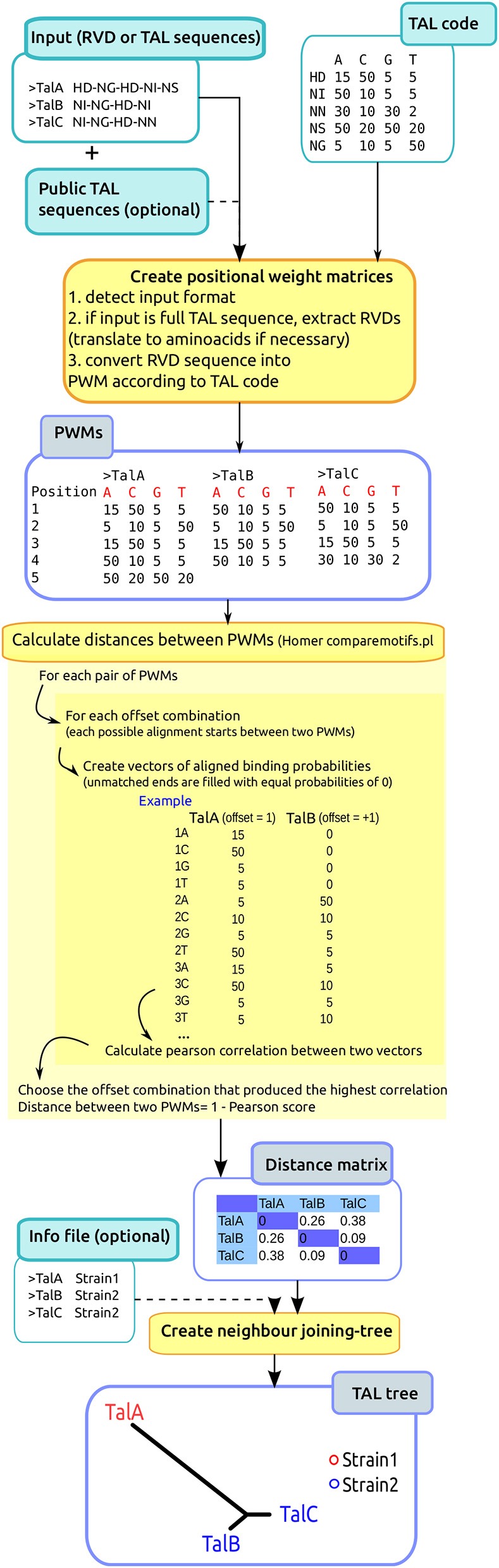
**FuncTAL workflow**. The diagram shows a summarized version of the FuncTAL workflow. An example is shown for three hypothetical TAL effectors with 4 or 5 RVDs. The values in the TAL code are the relative binding specificities for each nucleotide used by FuncTAL. Processes are shown in orange squares, inputs (lighter blue) and outputs are shown in blue squares.

TAL effectors act as transcription factors and their binding sites can be predicted according to a code (Boch et al., 2009; Moscou and Bogdanove, 2009; Noel et al., 2013). It is therefore feasible to compare the probable binding sites for TAL effectors using similar strategies as those devised to compare DNA motifs (Heinz et al., 2010). The program FuncTAL was designed to compare DNA binding capabilities for TAL effectors. Briefly, the program translates the RVD sequence of a TAL effector into a position weight matrix (PWM) stating the binding probabilities to nucleotides according to the TAL effector-DNA binding code (Boch et al., 2009; Moscou and Bogdanove, 2009). The PWMs are then compared using the strategy from the program HOMER (Heinz et al., 2010) to compare DNA motifs, which relies on calculating correlations for each position for two PWMs. The workflow for this program is depicted in Figure 5, and is explained in detail below.

#### Identification of RVDs and creation of PWMs

The program reads either a tabular file containing RVD sequences or, a FASTA file with nucleotide or amino acid sequences and then extracts RVDs. The sequence of RVDs for each TAL effector is then transformed into a position weight matrix according to a modified version of the RVD-nucleotide association matrix used by the program Talvez (Perez-Quintero et al., 2013) which was shown to perform well for the identification of known binding sites (Perez-Quintero et al., 2013). The matrix was modified to include updated RVD specificities according to the literature (Streubel et al., 2012; De Lange et al., 2013; Deng et al., 2014). The specificities are shown in Supplementary Table 3.

The program also builds and outputs a consensus binding site for each TAL effector by identifying the most probable nucleotide for each position. As with DisTAL, the user can choose whether to compare the input RVD or TAL effector sequences amongst each other or to include a set of RVD sequences available from public databases in the comparison.

#### Alignment and scoring of PWMs

To align and score the PWMs the program uses code from the script comparemotifs.pl of the HOMER suite (Heinz et al., 2010), which is designed to compare the binding sites of eukaryotic transcription factors.

The PWM comparisons are made for each pair of TAL effectors in the input. Every possible alignment between two matrices is evaluated by sliding the starting position (offset) of one matrix in respect to another. This process is unidirectional (i.e., the reverse or reverse complement of the binding sites are not compared), the alignments are ungapped, and the unmatched ends at either side of either matrix are filled with equal probabilities of matching any nucleotide.

For each offset, the Pearson correlation coefficient is calculated between two arrays A and B, where A is the ordered binding probabilities for each nucleotide and each position in one PWM, and B is the corresponding probabilities in a second PWM. The best alignment between the two PWMs is chosen by identifying the offset that produced the highest correlation. If x is the highest correlation between two PWMs, 1 – x is considered the distance between the matrices. A distance of 0 will correspond to TAL effectors that are identical in length and RVD sequence.

#### Creating trees of binding probabilities

The distances for each pair of TAL effector PWMs are organized into a matrix, which is then used to create a neighbor-joining tree using the R package APE (Paradis et al., 2004) in a user-defined format. As with DisTAL the tree tip labels can be colored using an additional input file from the user that contains the TAL effector IDs and categories used to group them (e.g., species or strain).

### FuncTAL accurately represents relations between functionally convergent TAL effectors

To show that FuncTAL can identify TAL effectors with unrelated RVD arrays but similar binding specificities, we decided to take advantage of three cases of experimentally observed functional convergence among TAL effectors.

**Figure 6:**
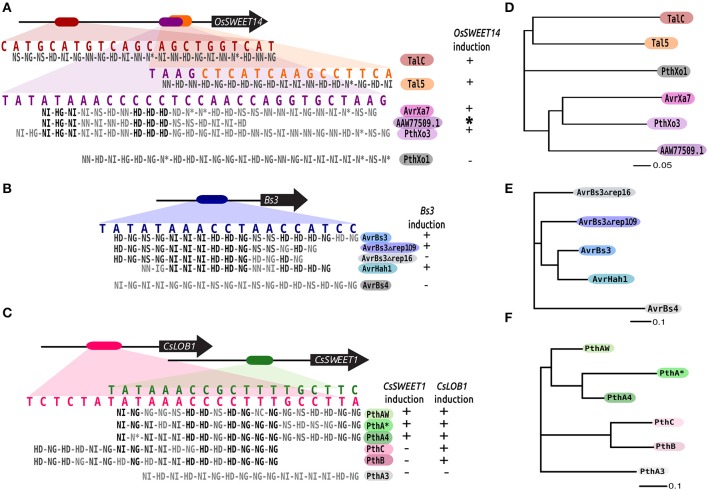
**Performance of FuncTAL with cases of functional convergence. (A–C)** Experimentally confirmed cases of functional convergence are shown for TAL effectors from *X. oryzae* pv. *oryzae* (TalC, AvrXa7, PthXo3, Tal5, AAW77509.1, and PthXo1) **(A)**, *X. euvesicatoria* (AvrBs3, AvrBs3Δrep16, AvrBs3Δrep109, and AvrBs4) and *X. gardneri* (AvrHah1) **(B)**, and *X. citri* pv. *citri* (PthA4, PthA^W^ and PthA^*^) and *X. citri* pv. *aurantifolii* (PthB and PthC) **(C)**. Targeted genes are shown as black arrows, and EBEs are depicted as colored boxes in the promoters. TAL effectors names are highlighted in a color panel corresponding to the color of the EBE they bind to. The 5′ region with a purple font in the EBE_Tal5_ corresponds to the 3′-end of the EBE_PthXo3_. TAL effectors RVD sequences are shown aligned to the corresponding EBEs, similar RVDs between TAL effectors are indicated by shading (darker RVDs are similar). + and – respectively indicate experimentally confirmed induction and absence of induction in the literature, * indicates prediction of binding without experimental confirmation. AvrBs4, PthXo1, and PthA3 were included as outgroups for each case, respectively. (**D–F)** Trees obtained by feeding the RVD sequences in **(A–C)** to FuncTAL with default parameters (phylogram layout), scale corresponds to FuncTAL scores.

One of the best-studied cases of functional convergence among TAL effectors is that of the rice *S* susceptibility gene *SWEET14* which is induced by multiple *X. oryzae* pv. *oryzae* TAL effectors targeting at least three different EBEs (Figure 6A). AvrXa7 and PthXo3, from strains PXO86 and PXO61 respectively, target overlapping EBEs in the *SWEET14* promoter (Yang and White, 2004; Chu et al., 2006; Antony et al., 2010). Another TAL effector from *Xoo* strain KACC10331, with similar RVDs but different length than AvrXa7 and PthXo3, is predicted to target the same site (accession number AAW77509.1 or YP_202894.1) (Perez-Quintero et al., 2013). Tal5 from *Xoo* strain MAI1 binds to another EBE in this promoter with minor overlap to that of AvrXa7/PthXo3 (Streubel et al., 2013), and TalC from *Xoo* strain BAI3 binds to an EBE with no overlap to the two other target sites (Yu et al., 2011). From this we expect that when fed to FuncTAL, TAL effectors that target completely overlapping sequences (AvrXa7, PthXo3 and the predicted AAW77509.1) will group together. As an outgroup, we included the TAL effector PthXo1 from *Xoo* strain PXO99^A^ known to target *SWEET11* which is another *SWEET* member acting as an *S* gene in rice (Yang et al., 2006). Indeed, using FuncTAL on these TAL effectors results in a tree where, AvrXa7, PthXo3, and YP_202894.1 are grouped together (Figure 6D). And although the EBE targeted by this group and that of Tal5 EBE overlap by 3 nucleotides, this is not enough for the program to consider them as functionally similar.

Another example of functional convergence is that of AvrBs3 from *X. euvesicatoria* strain 71–21 and AvrHah1 from *X. gardneri* strain XV444. These TAL effectors both bind to overlapping EBEs in the promoter of the pepper resistance gene *Bs3* (Schornack et al., 2006, 2008; Boch et al., 2009). Additionally, AvrBs3Δrep16 and AvrBs3Δrep109 are two artificial deletion derivatives of AvrBs3 (Herbers et al., 1992). When tested, it was found that AvrBs3Δrep16 lost the ability to bind to the AvrBs3 EBE in the *Bs3* promoter (Boch et al., 2009) (Figure 6B). AvrBs4, a TAL effector that activates the resistance gene *Bs4* was used as an outgroup (Schornack et al., 2004). When using FuncTAL on these TAL effectors, the resulting tree reflected the functional relation shown experimentally (Figure 6E).

Finally, another interesting case is that of a group of TAL effectors from *X. citri* (Figure 6C). PthA4, PthA^*^ and PthA^w^ originate from *X. citri* pv. *citri* strains IAPAR 306, X0053 and Xc270 respectively. These TAL effectors have somewhat similar RVD sequences, they bind to EBEs situated upstream of the *CsSWEET1* and *CsLOB1* genes, and induce their expression in sweet orange (Hu et al., 2014). Additionally, the *X. citri* pv. *aurantifolii* TAL effectors PthB and PthC effectively bind to an EBE in the *CsLOB1* promoter which is overlapping to that of PthA (Al-Saadi et al., 2007), however PthB and PthC fail to induce *CsSWEET1* (Hu et al., 2014). From this we expect all these TAL effectors to form a “functionally related” group with two subgroups: one comprising the PthA homologs and the other made of PthB and PthC. We thus fed the RVD sequences for these TAL effectors into FuncTAL. As an outgroup we included PthA3 which is a TAL effector from *Xcc* strain IAPAR 306 that fails to induce either *CsLOB1* or *CsSWEET1*. The resulting tree reflected the expected relations (Figure 6F).

In these analyses the maximum pairwise distances for TAL effectors binding overlapping EBEs were 0.70 in the *X. citri* TAL effectors (between PthC and PthA^w^), 0.67 in the *X. oryzae* TAL effectors (between AAW77509.1 and AvrXa7) and 0.44 in the *Bs3*-targeting TAL effectors (between AvrHah1 and AvrBs3Δrep109). Ideally, this data would serve to establish thresholds to group TAL effectors with functional convergence. However, these values might be too variable to make accurate recommendations. More experimental data will be needed to accurately define these thresholds. Meanwhile, FuncTAL distances below 0.5 may be an adequate suggestion to consider TAL effectors as functionally similar.

### FuncTAL and DisTAL show different groupings of TAL effectors

**Figure 7:**
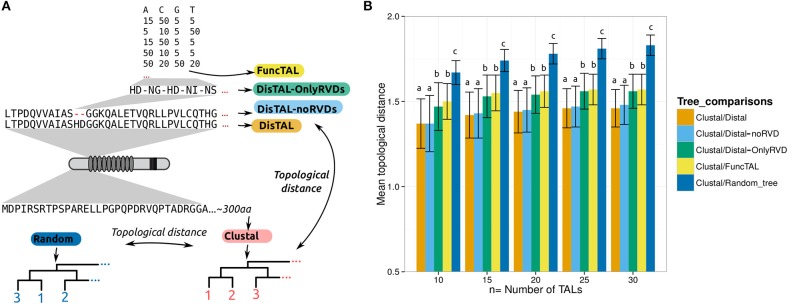
**Comparison of trees obtained with FuncTAL and DisTAL using different parameters**. Trees were obtained by running the programs of the QueTAL suite with different parameters using different sets of randomly selected TAL effectors. Reference trees were generated with ClustalW using the N-terminal region of the TAL effectors and randomly-generated trees were used as negative controls. **(A)** Diagram showing the features of a TAL effector sequence that was used for each treatment: PWMs obtained from RVD sequences for FuncTAL, RVD sequences for Distal-OnlyRVDs, repeats without RVDs for DisTAL-noRVDs, full repeats for DisTAL (default), and N-terminal for ClustalW. *z*, number of repeats in a TAL effector; n, number of randomly selected TAL effectors from our dataset used to build the trees. **(B)** Topological distance between the trees obtained with each treatment and those obtained with ClustalW using the N-terminal region for sets comprised of different numbers of TAL effectors, each bar represents the mean obtained for 100 sets, error bars indicate standard deviation. The topological distance was normalized by dividing by the number of nodes in the tree. Lowercase letters on top of the bars indicate groups with equal means as determined by two-tailed Wilcoxon tests (*p* > 0.05).

To assess how the results from DisTAL and FuncTAL differ from each other based on different settings we followed an approach based on the comparison of topological distances. For this, a set of *n* complete TAL amino acid sequences was selected at random from our dataset and five trees were created for that set with the following methods (Figure 7A):

- DisTAL using default parameters (using the full repeat),
- DisTAL excluding RVDs,
- DisTAL using only RVDs,
- FuncTAL using default parameters,
- ClustalW alignment and neighbor-joining phylogenetic tree using only the N-terminal region, to be used as a reference.

The topological distance was calculated as before between each tree and the one obtained with the N-terminal region using ClustalW. As a negative control, the trees were also compared to a random tree [using rtree from the R package APE, (Paradis et al., 2004)]. The process was repeated 100 times for different values of **n**. As a result, the trees obtained with DisTAL using either the full repeats or excluding the RVDs were the most similar to the N-terminal reference (Figure 7B), suggesting that these methods infer a phylogeny similar to that obtained using a more traditional approach. Yet, the average normalized distance between each of the two methods when compared to ClustalW (N-terminal) was higher than 1. This indicates that at least half the nodes in the trees differed from each other, suggesting that there is different information in the repeat sequences to that in the N-terminal region.

When compared to each other, the trees obtained with DisTAL with or without the RVDs also had a relatively high topological distance (mean = 1.25 when *n* = 20). This difference between the trees may be explained by RVDs being under different selective pressure (related to target sequence specificity) than the rest of the repeat sequence (which is probably under selective pressure for protein conformation). Also, the mean topological distance was higher when comparing the N-terminal trees to those obtained with FuncTAL or with DisTAL using only RVDs (Figure 7B). This indicates that the information contained in RVD sequences is somewhat different from that in the rest of the protein, thus, binding similarities are expected to not necessarily follow the phylogeny due to the selection for them to bind a specific sequence element in the host genome.

**Figure 8:**
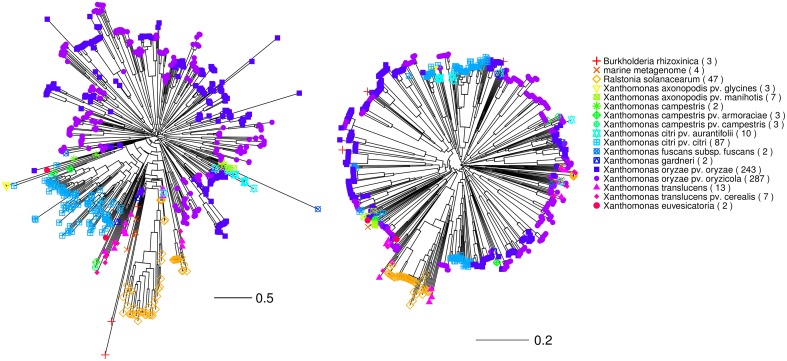
**Trees obtained for a large set of TAL effectors using DisTAL and FuncTAL**. DisTAL (left) and FuncTAL (right) were used to build trees (fan layout) for a set of 725 TAL effector sequences from 18 different taxonomic groups. The group of each TAL effector is indicated by the tip colors in the tree. The “marine metagenome” TAL effectors correspond to TAL effector sequences from an unidentified organism (Juillerat et al., 2014). Scales are shown below the trees, these are not comparable between the programs due to the methods used.

Finally, we ran our complete set of 725 TAL effector sequences through DisTAL and FuncTAL (default parameters), and compared the distribution of taxonomic groups. As seen in Figure 8, the tree obtained with DisTAL seems to follow at least partially the expected phylogeny of the groups analyzed. For example, the TAL and RipTAL proteins from respectively *X. citri* pv. *citri* and *Ralstonia solanacearum* form discrete well-defined groups. In contrast, the TAL effectors from the two main pathovars of the species *X. oryzae* appear distributed in many clusters. Additionally, the recently discovered TAL effectors-like proteins from an unknown marine organism identified in metagenomic data (Juillerat et al., 2014) as well as those of *Burkholderia rhizoxinica* (De Lange et al., 2014; Juillerat et al., 2014) appear as separated from the *Xanthomonas* TAL effectors and closer to the *R. solanacearum* RipTAL proteins.

On the other hand, the tree obtained by FuncTAL shows that clusters of “functionally similar” TAL effectors often include sequences coming from different taxa (Figure 8). However, TAL effectors from certain clades seem to have very specific clustering, particular examples of this are the *R. solanacearum* RipTAL proteins as well as the TAL effectors from *X. translucens* to some extent, that form clusters in the tree that are distinct from the other clades. This might be due to specific RVD usage in these groups. Indeed RipTAL proteins are predicted to bind to G-C rich DNA regions in contrast to the A-T rich regions predicted for most TAL effectors (De Lange et al., 2013). Naturally occurring targets for these effectors are yet to be confirmed.

Altogether these results show that DisTAL and FuncTAL display different but complementary information that can be used to infer evolutionary relationships between taxons and predict cases of functional convergence between TAL effectors.

## Discussion

In order to understand how TAL effectors or other related proteins differ from each other within and between strains of one or several pathovars, current approaches mainly rely on the evaluation of genetic distances through the alignment of the N-terminal and/or C-terminal regions, thus excluding the central region due its repetitive nature. To fill this gap, the first aim of this work was to adapt existing methods to compare the sequences of TAL effectors repeats and infer evolutionary scenarios. The program DisTAL, used to calculate phylogenetic distances, relies on the hypothesis that one of the major driving forces of evolution of TAL effectors is probably through recombination between repeats or slipped strand mispairing during DNA replication, resulting in duplication, deletion or reorganization of one or several repeats. This hypothesis is supported by the fact that in several strains the TALome appears to be the result of numerous duplications (e.g., in *Xoo* strain PXO99^A^ (Bogdanove et al., 2011), that deletions occur in nature (Vera Cruz et al., 2000), and that internal recombination events were detected upon experimental evolution assays (Yang et al., 2005) and for TALEN systems *in vitro* (Lau et al., 2014). Since the structure of the genes and the mechanisms of evolution are expected to be similar to that of microsatellites, we chose to adapt an algorithm and program designed to compare coded “maps” representing tandem repeats (Abouelhoda et al., 2010).

DisTAL considers repeats as evolutionary units and finds similarities between arrays of repeats. Using simulated data we showed that the program can accurately infer relationships between arrays of repeats derived from one ancestor that underwent processes of insertions, deletions and replacement (mutation) of repeats, performing better than a traditional multiple alignment methods. Possible caveats of the method include the fact that duplication breakpoints might not correspond to the way the repetitions have been traditionally defined, though so far there is not enough data to accurately pinpoint where these events occur. A possible workaround this problem that we will try to implement in future versions of DisTAL is to adapt a method that does not restrict tandem repeats by unit boundaries like a graph-based applied to study LRR tandem units in GALA effector proteins from *R. solanacearum* (Szalkowski and Anisimova, 2013).

The DisTAL parameters for penalizations for insertions, deletions and duplications to be used by the ARLEM algorithm were optimized by finding short alignments with low variability in their scores for TALomes of fully sequenced strains. These parameters may not accurately reflect the rate at which these events occur. Studies are needed where the evolution of TAL effectors is followed on natural bacterial populations for which short term evolutionary patterns are known. Alternatively, mutagenesis or artificial evolution experiments on TAL effectors would also be a great resource to understand variation in these proteins, in a similar way as to what has been done to create TAL effector variants with reduced virulence (Yang and White, 2004) or in the way mutational events were studied in viral vectors carrying TAL repeats (Lau et al., 2014). These types of experiments will also help determine the recombination points on these proteins.

So far, DisTAL uses amino acid sequences for all of the comparisons instead of nucleotides because the former are shorter, reducing greatly the computational time to calculate distances. This could represent a loss of information since synonymous mutations are not taken into account. However, this loss may be minor since, for example, a set of 169 complete and unique nucleotide TAL repeat sequences (from public available databases) corresponds to 168 unique amino acid sequences.

A robust scientific framework to understand and anticipate TAL effector diversity, evolution and dynamics is essential to assess the value of control strategies based on manipulation of their host targets (Boch et al., 2014). DisTAL is the first program to allow classification of TAL effectors in a manner which includes the possibility of repeat rearrangement and duplication as a major determinant of TAL effectors evolution. The program includes pre-processing of any TAL sequence, and alignment of repeat sequences based on the ARLEM program (Abouelhoda et al., 2010). We believe this tool not only is more reliable at comparing and classifying TAL effectors according to their phylogeny but will also offer precious help for future experimental and modeling works on TAL effector evolution.

An important feature of TAL effectors is that their function can, to some extent, be predicted from their RVDs sequence thanks to their modular and specific interaction with DNA (Boch et al., 2009; Moscou and Bogdanove, 2009). Indeed tools already exist to predict candidate EBEs in plant genomes (Doyle et al., 2012; Grau et al., 2013; Perez-Quintero et al., 2013). As more TAL effectors are discovered, notably through sequencing of entire TALomes (e.g., Wilkins et al., 2015), it is essential to classify them according to what can be hypothesized about their function. The second main output of this study is the design of a tool for comparing TAL effectors through their EBEs, which will facilitate the identification of cases of functional convergences and therefore candidate susceptibility hubs. FuncTAL calculates correlations between potential TAL effector binding sites by translating RVD sequences into PWMs according to the RVD-DNA code. The program successfully inferred functional relations for known cases of functional convergence among TAL effectors targeting overlapping EBEs. Notably it associated TAL effectors that have very different RVD sequences and for which convergence would normally be difficult to predict (i.e., the association between PthB and PthC to the PthA group).

For now, the program does not take into account binding specificities not encoded by RVDs, such as those for position 0 in the EBE. For *Xanthomonas* TAL effectors, a thymine (T_0_) preceding the EBE is required in most cases for binding and activity (Boch et al., 2009) whereas for *Ralstonia* RipTAL effectors, a guanine is required instead (De Lange et al., 2013). Because these requirements are encoded by the degenerated -1 repeat situated upstream of the central repeats, binding is not determined by RVDs but rather by the overall structure of this region (Mak et al., 2012). The specific features in the -1 repeat determining the preference for different nucleotides have yet to be identified. Structure studies suggest that in *Xanthomonas* TAL effectors, binding to T_0_ is coordinated by a tryptophane (W232) in the -1 repeat (Mak et al., 2012). However, repeat number and RVD-composition seem to also affect the specificities at position zero (Schreiber and Bonas, 2014). A future version of the program may account for position zero specificity once it is possible to predict it from the TAL effector sequence and calculate binding probabilities from it.

So far the alignments made with FuncTAL are ungapped because TAL effectors bind to DNA in a sequential manner, with one RVD corresponding to one base pair, without gaps. A possible exception to this rule are TAL effectors that contain aberrant or longer than normal repeats, that have been shown to allow flexibility in binding and tolerating short gaps in their corresponding EBE (Richter et al., 2014). However, biological examples for this type of flexibility are rare, the only TAL effectors with aberrant repeats for which binding has been extensively studied are AvrXa7 and PthXo3 (Richter et al., 2014). Once the exact mechanisms of aberrant repeat binding specificities are described they may be included in the program.

It is worth stressing that FuncTAL does not use promoter sequences to infer the relations. This means that cases of functional convergence where the EBEs are not overlapping, such as TalC and AvrXa7 EBEs in rice, will be impossible to predict using these method. Yet, FuncTAL can be of interest to follow the evolution of TALomes in epidemics, notably under selective pressures, e.g., when pathogen populations are constrained by host varieties carrying resistance genes such as recessive loss-of-susceptibility alleles or dominant *R* genes. As for the analysis of the full TAL effectors dataset corresponding to 18 different taxonomic groups, the associations found by FuncTAL are more likely reflective of RVD usage and general binding preference than actual potential for convergent gene induction. We expect that to be the case for the well-defined group found for *R. solanacearum* and *X. translucens*. Indeed, the effectors in this group may have different targets, but their association reflects a preferential targeting for a certain type of sequences (GC rich regions), which may be of biological relevance.

Developing methods to predict true evolutionary and functional convergence is still needed, particularly since TAL effectors tend to preferentially target specific genes or gene families that are crucial for disease development [Reviewed in Hutin et al. (2015), and elsewhere (Boch et al., 2014)]. Future work will be aimed at predicting these relations by combining expression data, binding site prediction and distances generated with the methods presented here.

Here we obtained trees with DisTAL and FuncTAL from a large set of TAL effector (and related) proteins showing in some cases well-defined groups that often coincide with the species or pathovar phylogeny. In the future, it will be of interest to go more in detail in the analysis of some of these groups and scrutinize which relations arise between particular strains. In particular, it is worth noting that for a few *TAL* effector genes, *Xoo* and *Xoc* orthologs seem closer than to any of their paralogs. This contrasts with results obtained upon alignment of the N- and C-termini regions of some of these TAL effectors, showing *Xoc* and *Xoo* TALomes to cluster separately (Bogdanove et al., 2011; Yu et al., 2011). Such contrasted results potentially highlight DisTAL's higher accuracy to infer phylogenetic relations, notably because it relies on information coming from the central repeat region. It would also be of interest to evaluate the nature and the function of these “conserved” TAL effectors, knowing that a few rice genes are known to be targeted by both pathovars (Cernadas et al., 2014).

We expect this suite to be a constantly expanding project. Other than the possible improvements mentioned above we expect to be able to add new functionalities and features to the suite including: (1) a way to use TAL effector distances obtained from either FuncTAL or DisTAL to calculate similarities between strains with fully sequenced TALomes, (2) a tool to find over-represented strings of RVD or repeat sequences in TAL effectors that may constitute functional evolutionary units, (3) tools to compare TAL effectors binding sites to plant transcription factor binding sites (aiming to help in genetic engineering strategies where resistance against bacteria is to be achieved by mutating EBEs without altering endogenous regulation of genes).

In conclusion, this work provides a more accurate tool for inferring genetic distances between *TAL* effector genes through the use of the phylogenetic information encoded by the repeat region. It also offers the possibility to classify groups of TAL effectors with similar DNA-binding specificities, i.e., targeting the same EBEs, thereby highlighting cases of functional convergence on key susceptibility genes. Such information can be precious when dealing with a high number of candidate host targets from which a selection has to be made to choose for the best S genes candidates. Overall in the present context where a relentless flow of TALomes and host genomes are made available through next-generation sequencing methods, we hope the QueTAL suite will be helpful to push forward our understanding of TAL effectors evolution and functional diversity.

## Author contributions

AP, designed the programs and performed the analyses, LL, designed the web platform for the programs, JG and AE devised the strategy for DisTAL, SC, helped in the design of the program and the validation strategies, BS and LG directed the work, BS, LG, and AP wrote the manuscript.

### Conflict of interest statement

The authors declare that the research was conducted in the absence of any commercial or financial relationships that could be construed as a potential conflict of interest.

## Supplementary material

Supplementary Figure 1 **Indel and duplication penalization for DisTAL**. Variation in alignment length and score values for TAL effectors from *X. citri pv. citri* IAPAR 306 using Distal with different indel and duplication penalization values. Each point represents a pairwise alignment between two TAL effectors, red lines indicate range between 5 and 10. Please redefine what parameters alpha and beta refer to.

Supplementary Figure 2 **Variation in alignment length and score values for TAL effectors from strains *Xoo* PXO99^A^ (18 TAL effectors) and *Xoc* BLS256 (28 TAL effectors) using Distal with different indel and duplication penalization values**. Each point represents a pairwise alignment between two TAL effectors, red lines indicate range between 5 and 10.

Supplementary Figure 3 **DisTAL performance with *in silico*-evolved TAL effectors**. Sets of eight TAL effectors (named A–H) resulting from simulated evolution were fed into DisTAL and ClustalW, the resulting trees were compared to the expected tree [((A B)(C D))((E F)(G H))], the scatter plot shows the topological distance. Different values of alpha (probability of repeat replacement) and beta (probability of repeat indel) were used to generate the sets of TAL effectors. Each point represents the average topological distance for 100 sets of TAL effectors, error bars indicate standard deviation.

Supplementary Table 1 **Accession number and RVD sequences of publicly available TAL effector sequences**.

Supplementary Table 2 **Species composition of the full dataset of TAL effector sequences used in this work, including public sequences and those donated by collaborators**.

Supplementary Table 3 **RVD-DNA specificities used by FuncTAL to construct PWMs**.

## Abbreviations

EBE: Effector binding element
Gb: Gigabyte
GHz: Giga hertz
indel: insertion/deletion
LRR: Leucine-rich repeat
PWM: Positional weight matrix
RVD: Repeat variable di-residue
TAL: Transcription activator-like
Xoc: Xanthomonas oryzae pv. oryzicola
Xoo: Xanthomonas oryzae pv. oryzae.
