# Supplementary figures and images for: QueTAL: a suite of tools to classify and compare TAL effectors functionally and phylogenetically
Source: Front Plant Sci. 2015 Aug 3;6:545. doi: 10.3389/fpls.2015.00545 (PMC4522561; doi:10.3389/fpls.2015.00545)

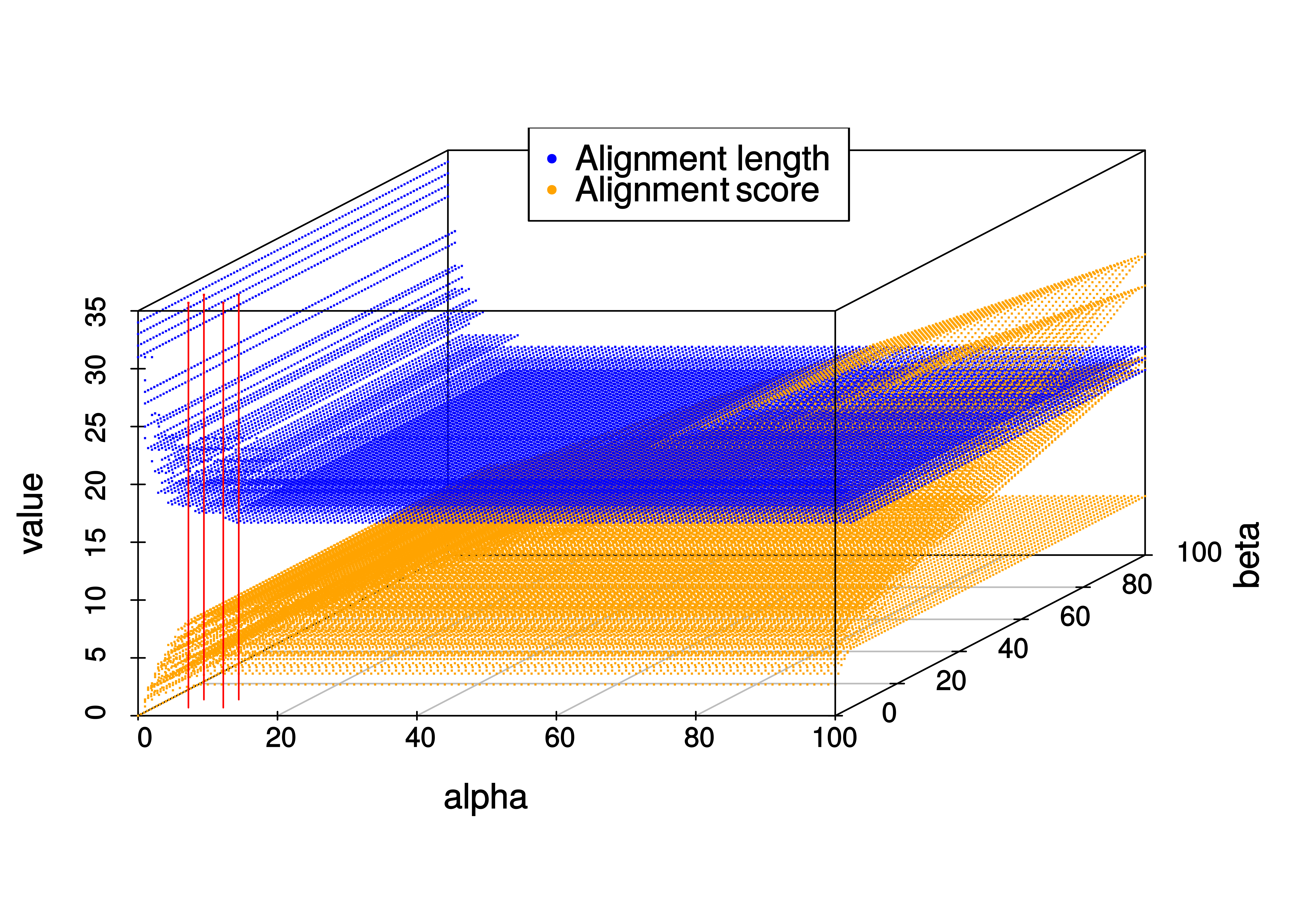

Supplement: Supplementary Figure 1 — Indel and duplication penalization for DisTAL. Variation in alignment length and score values for TAL effectors from X. citri pv. citri IAPAR 306 using Distal with different indel and duplication penalization values. Each point represents a pairwise alignment between two TAL effectors, red lines indicate range between 5 and 10. Please redefine what parameters alpha and beta refer to. [file Image1.TIFF]

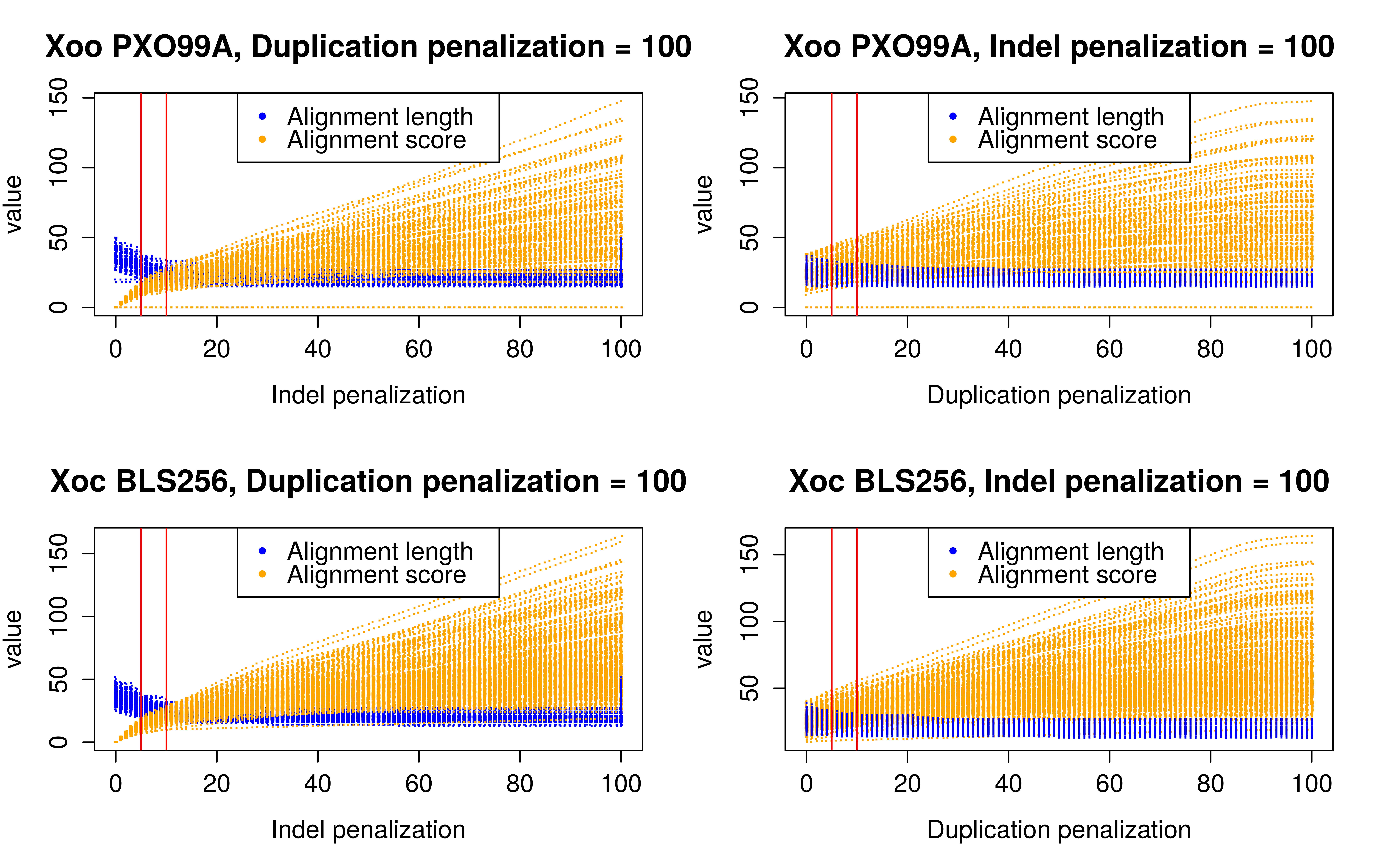

Supplement: Supplementary Figure 2 — Variation in alignment length and score values for TAL effectors from strains Xoo PXO99A (18 TAL effectors) and Xoc BLS256 (28 TAL effectors) using Distal with different indel and duplication penalization values. Each point represents a pairwise alignment between two TAL effectors, red lines indicate range between 5 and 10. [file Image2.TIFF]

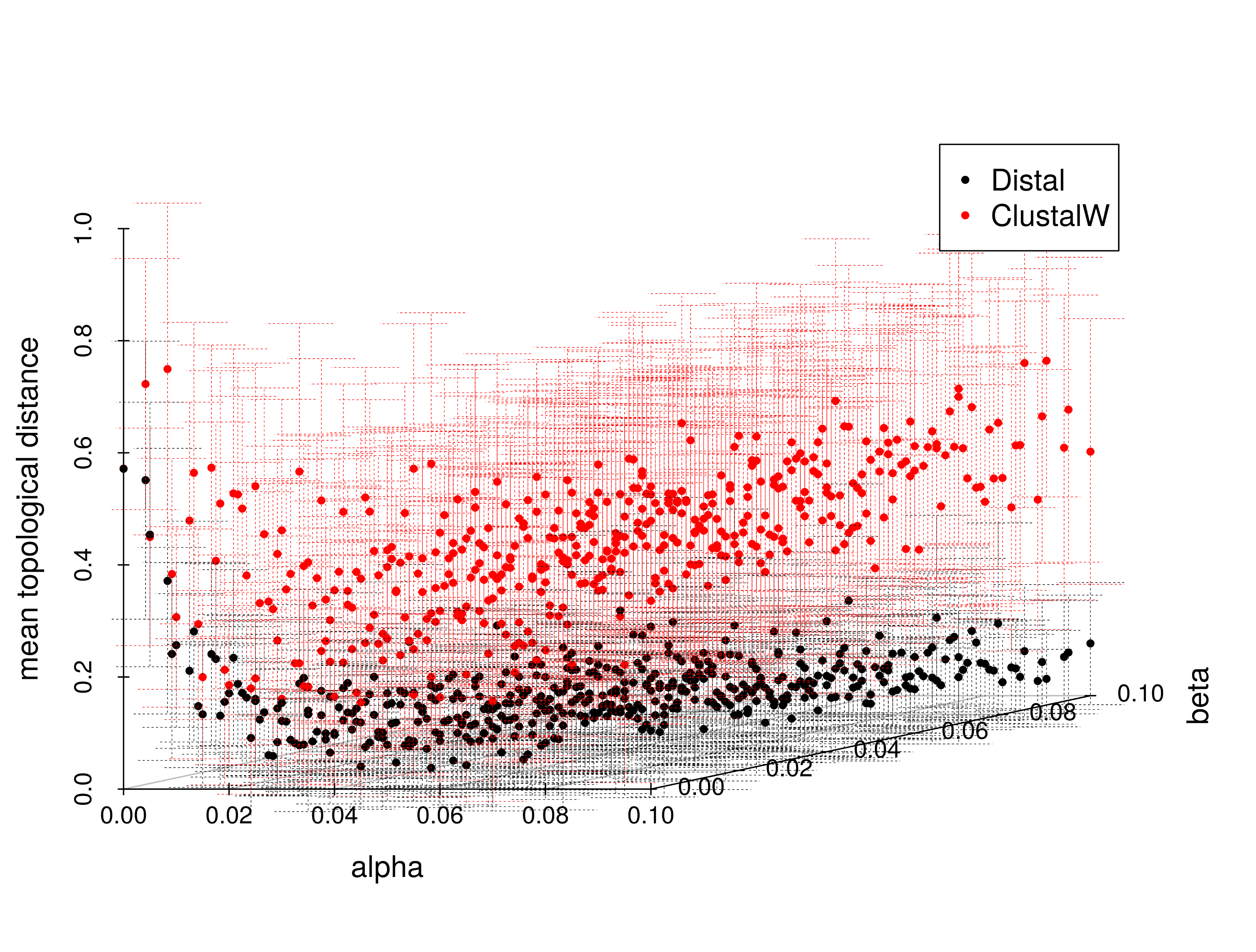

Supplement: Supplementary Figure 3 — DisTAL performance with in silico-evolved TAL effectors. Sets of eight TAL effectors (named A–H) resulting from simulated evolution were fed into DisTAL and ClustalW, the resulting trees were compared to the expected tree [((A B)(C D))((E F)(G H))], the scatter plot shows the topological distance. Different values of alpha (probability of repeat replacement) and beta (probability of repeat indel) were used to generate the sets of TAL effectors. Each point represents the average topological distance for 100 sets of TAL effectors, error bars indicate standard deviation. [file Image3.TIFF]
